# Health Benefits of Heat-Killed (Tyndallized) Probiotics: An Overview

**DOI:** 10.3390/ijms20102534

**Published:** 2019-05-23

**Authors:** Núria Piqué, Mercedes Berlanga, David Miñana-Galbis

**Affiliations:** 1 Microbiology Section, Department of Biology, Healthcare and Environment, Faculty of Pharmacy and Food Sciences, Universitat de Barcelona (UB), Av Joan XXIII, 27-31, 08028 Barcelona, Catalonia, Spain; mberlanga@ub.edu (M.B.); davidminyana@ub.edu (D.M.-G.); 2 Institut de Recerca en Nutrició i Seguretat Alimentària de la UB (INSA-UB), Universitat de Barcelona, 08921 Barcelona, Catalonia, Spain

**Keywords:** probiotics, heat-killed, tyndallized, Lactobacillus, Bifidobacterium, immunomodulatory

## Abstract

Nowadays, the oral use of probiotics is widespread. However, the safety profile with the use of live probiotics is still a matter of debate. Main risks include: Cases of systemic infections due to translocation, particularly in vulnerable patients and pediatric populations; acquisition of antibiotic resistance genes; or interference with gut colonization in neonates. To avoid these risks, there is an increasing interest in non-viable microorganisms or microbial cell extracts to be used as probiotics, mainly heat-killed (including tyndallized) probiotic bacteria (lactic acid bacteria and bifidobacteria). Heat-treated probiotic cells, cell-free supernatants, and purified key components are able to confer beneficial effects, mainly immunomodulatory effects, protection against enteropathogens, and maintenance of intestinal barrier integrity. At the clinical level, products containing tyndallized probiotic strains have had a role in gastrointestinal diseases, including bloating and infantile coli—in combination with mucosal protectors—and diarrhea. Heat-inactivated probiotics could also have a role in the management of dermatological or respiratory allergic diseases. The reviewed data indicate that heat-killed bacteria or their fractions or purified components have key probiotic effects, with advantages versus live probiotics (mainly their safety profile), positioning them as interesting strategies for the management of common prevalent conditions in a wide variety of patients´ characteristics.

## 1. Introduction

Currently, there is no doubt that the influence of the intestinal state on the health is gaining great interest, not only among the scientific community [1,2,3,4], but also for patients and consumers who are willing to adapt their diet habits to achieve the best well-being and health, besides other animal- or environment-related motives [5].

In this regard, gut microbiota, defined as a complex and dynamic microbiome containing more than 1000 different species, characteristic for each gastrointestinal (GI) tract segment, is recognized to be indispensable for homeostatic physiological functions in human health [1,3] at both the intestinal and extra-intestinal levels.

With the advent of new sequencing technologies, mainly based on the 16S ribosomal RNA genes, and the development of sophisticated bioinformatic tools, characterization of gut microbiota is being advanced, leading to the understanding of the composition and function of bacterial populations throughout the intestine and to the influence of fluctuations in the diversity of gut bacterial populations (known as dysbiosis) in the development of diseases [1,4,6,7].

This knowledge has been translated in a great interest in those therapeutical strategies to directly or indirectly influence gut microbiota to obtain clinical benefits, such as the use of probiotics, prebiotics, and other food supplements or fecal transplantations [8,9,10].

Probiotics, according to the revised definition of Food and Agriculture Organization (FAO)/World Health Organization (WHO, are considered as non-pathogenic live microorganisms that, when administered in adequate amounts, confer a health benefit on the host. Now probiotics are widely used in many countries in clinical practice and, frequently, are acquired by consumers with or without prescription [9,11,12,13,14]. In most cases, probiotics, mainly strains of *Bifidobacterium* or *Lactobacillus* species, come from the gut microbiota of healthy humans or from dairy products [15]. Probiotics also include species from the genera *Streptococcus*, *Bacillus*, and *Enterococcus* and the yeast *Saccharomyces*, which has been used as probiotics for many years [11,16,17].

Among the main effects of probiotics at the intestinal level, the following are noteworthy: Balancing and restoration of the gut microbiota, protection against pathogens, immunomodulation, and maintenance of intestinal barrier integrity [18]. Probiotics are widely used in dietary supplements, food, infant formula formulations, and medical devices [19,20]. They have demonstrated significant potential as therapeutic options for a variety of diseases, mainly gastrointestinal diseases (including acute infectious diarrhea, antibiotic-associated diarrhea, ulcerative colitis, irritable bowel syndrome, functional gastrointestinal disorders, or necrotizing enterocolitis), but also extra-intestinal disorders, such as hepatic encephalopathy [9,21].

However, there are still many issues on the table, for example, safety issues with the use of live microorganisms particularly in vulnerable populations [14,22,23,24], the lack of clear clinical recommendations in each specific clinical situation [9,21], the lack of compelling evidence from clinical trials for certain indications [8], the limited regulation of probiotics [20], or the lack of studies assessing the viability of microorganisms once in the intestine and the differences between viable or non-viable microorganisms [25].

In a recent survey on probiotic-prescribing practices among health care providers and review of current guidelines and published large clinical trials, it was concluded that recommendations appear to be inconsistent, non-specific, and, frequently, upon patient request. In a significant proportion, the choice of probiotic was left to the patient or the pharmacist. The three most common clinical indications for probiotics were prevention and treatment of antibiotic-related side effects and irritable bowel syndrome [21].

Moreover, safety issues with the use of live strains have been arisen in certain patient groups, such as neonates [11,26] and vulnerable patients [23], particularly due to translocation of bacteria from the gut to the systemic circulation, leading to an increased interest to use non-viable heat-killed probiotics [14,22,27].

There is considerable published evidence that preparations containing dead cells and their metabolites can also exert relevant biological responses, restoring the normal intestinal homeostasis, in many cases similar to that seen with live cells, although with potential differences [22,27]. After inactivation of bacteria, mainly by heat treatment, dead cells can release bacterial components with key immunomodulating effects and with antagonizing properties against pathogens. Different bacterial components, such as lipoteichoic acids, peptidoglycans, or exopolysaccharides (EPS), have been proposed to be mainly involved in these properties in preparations containing heat-killed bacteria [27,28,29].

Favorable properties of heat-killed bacteria have been observed in in vitro, animal models [27], and clinical trials [30,31], which have demonstrated their benefits in different indications, for example in neonates, without incurring the risks associated with live microorganisms [14,32], and with pharmaceutical advantages in terms of transport and storage (Table 1).

The objective of this article is to review the recent published studies indicating that heat-killed bacteria with health benefits can be a safe and feasible strategy for the management of different diseases, mainly gastrointestinal disorders, focusing on the possible mechanisms of action involved, in comparison with live strains.

## 2. Beneficial Effects of Probiotics

The most extensively-studied and widely-used probiotic bacteria are *Lactobacillus* and *Bifidobacterium* [14,33,34,35]. Species of these two genera (including *Bifidobacterium breve*, *Bifidobacterium longum*, *Lactobactillus fermentum*, *Lactobacillus plantarum*, *Lactobacillus casei* or *Lactobacillus rhamnosus*) naturally inhabit the human gastrointestinal tract, and are thought to play pivotal roles in maintaining human health [34,36]. Therefore, the oral administration of probiotics is thought to reinforce the physiological functions of gut microbiota at the intestinal level.

**Figure 1:**
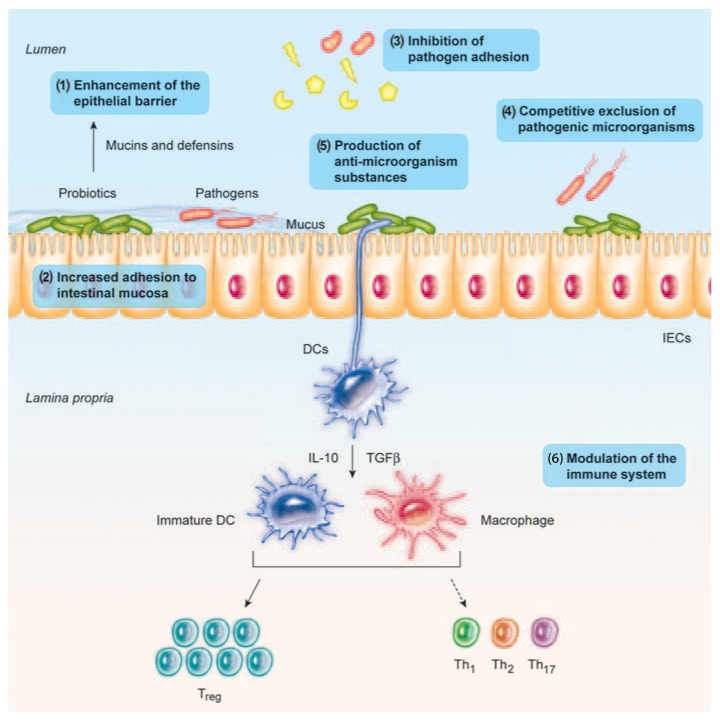
Main mechanisms of action of probiotics [19].

Probiotics are able to fight against pathogens by producing antimicrobial compounds and decreasing pH (with lactic acid production), and competing with pathogens for adhesion and colonization, and for nutrients and other growth factors in the gut [35], and suppressing the growth of pathogenic bacteria by directly binding to Gram-negative bacteria [9,19] (Figure 1).

Moreover, a body of evidence demonstrates that probiotic bacteria have immunomodulating properties, regulate inflammation in a number of ways, and enhance the epithelial barrier function to prevent chronic inflammation in the gut [19,35] (Figure 1). At the intestinal level, probiotics can also upregulate the intestinal electrolyte absorption and exert an effect on gut motility and constipation [9,37].

### 2.1. Immunomodulating Effects of Probiotics

There is evidence indicating that probiotics have immunomodulatory properties and protect against infection [33,38]. Probiotics, in strain-specific and dose-dependent manner, can modulate nonspecific cellular immune responses mediated by macrophages, natural killer (NK) cells, antigen-specific cytotoxic T-lymphocytes, and the release of cytokines [33].

Enhancement of innate immune responses has also been described for most probiotic strains, with IL-12 production by antigen-presenting cells (APCs), and the subsequent activation of host NK cells and promotion of type 1 helper T (Th1) cell responses [33,38].

Probiotics also enhance acquired immunity and induce IgA secretion in the intestine [38] by different mechanisms [39], with the activation of B lymphocytes and T cells [33,39].

However, the precise molecular mechanisms underlying the effects of each probiotic strain and different combinations of probiotic bacteria at the different immune pathways have not been fully resolved [38]. It should be also taken into account that, in formulations containing different live probiotics, certain species could inhibit the stimulatory effect of others [33,40].

Differences in the degree of stimulation of the defenses have been reported among different probiotic strains and different probiotic combinations, thus supporting the research on the active bacterial cellular components causing the variable immune stimulation [40], and identifying the levels at the intestinal level at which they can interact (outer, inner mucus layer, epithelium, immune cells).

#### 2.1.1. Anti-Inflammatory Responses

Probiotic bacteria confer anti-inflammatory responses by modulating different signaling pathways [35,41]. Different anti-inflammatory effects at the intestinal level have been described with probiotics, for example, enhancement of the epithelial barrier function in the gut [19,35,42]; attenuation of barrier dysfunction due to pro-inflammatory cytokines [43]; or modulation of intestinal anti-inflammatory responses such as the expansion of the T-regulatory response, which may be relevant for its use in chronic inflammatory disease [41,44].

Probiotic lactobacilli and bifidobacteria have been shown to down-regulate the production of inflammatory mediators (such as IL-6 and TNF-α) upon exposure to pro-inflammatory compounds such as lipopolysaccharide (LPS) in intestinal epithelial cells [35,45,46] and also beyond the gut (reduction of pro-inflammatory biomarkers in plasma from patients treated with *Bifidobacterium infantis*) [47].

#### 2.1.2. Enhancement of the Epithelial Barrier Integrity

The intestinal barrier is a major defense mechanism, including the mucous layer, antimicrobial peptides, secretory IgA, and the epithelial junction adhesion complex, with the aim to maintain epithelial integrity and to protect the organism from the environment, including pathogens [19].

Probiotic bacteria have been studied for their involvement in reinforcing the intestinal barrier, although the mechanisms involved are not fully elucidated [19,48]. Probiotics may initiate repair of the barrier function after damage induced by different pathological conditions, such as *E. coli*-induced mucosal disruption [19,49]. Probiotics can also prevent the cytokine-induced epithelial damage, which is characteristic of inflammatory bowel disease [19,50].

On the intestinal barrier, some strains can also block pathogen entry into the epithelial cells by increasing the mucus barrier, by stimulating the release of mucin granules from Goblet cells; and by maintaining the intestinal permeability, by increasing the intercellular integrity of apical tight junctions [19,51,52].

#### 2.1.3. TLR-2 Receptor

Toll-like receptors (TLR) are a family of 11 transmembrane proteins (TLR-1-TLR11) expressed on various immune and non-immune cells that recognize specific patterns of microbial components and regulate the activation of both innate and adaptive immunity [19]. The existence of several TLRs enables the innate immunity to recognize different groups of pathogens, while initiating appropriate and distinct immunological responses [53]. In humans, TLR1, TLR2, TLR4, TLR5, TLR6, and TLR10 primarily respond to bacterial surface-associated pathogen-associated molecular patterns (PAMPs) [19].

Several studies have demonstrated that Toll-like receptor-2 (TLR2) is required for probiotic strains to exert their immunomodulatory and anti-inflammatory effects [19], while stimulation of TLR4 can induce potent inflammatory responses [53].

Stimulation of TLR2 is particularly important for regulating inflammatory signaling pathways for Gram-positive bacteria [35,45] and has an important role in enhancing transepithelial resistance to invading bacteria [19,54]. Conversely, mutations in the TLR2 gene appear to be associated to severe inflammatory bowel disease [54,55]. While TLR4 recognizes Gram-negative bacteria components, such as LPS, TLR2 recognizes cell wall components of Gram-positive bacteria, including probiotic bacteria [54].

Therefore, stimulation of TLR2 by probiotic bacteria can be one of the keys for the favorable effects of probiotics at the intestinal level, leading to anti-inflammatory states that enhance the intestinal barrier integrity.

#### 2.1.4. NLRP3 Inflammasome

Nucleotide-binding oligomerization domain-containing protein (NOD)-like receptors (NLRs) are also known to transmit signals upon interaction with gut microbiota [19]. In particular, NLRP3 inflammasome is important to the maintenance of epithelial integrity [56] and the defense against pathogen infection in the intestine [57].

### 2.2. Protective Effects against Pathogenic Bacteria

Gut microbiota has a relevant role fortifying the epithelial barrier against enteric pathogens [58]. Probiotics including *Lactobacillus* and other lactic acid bacteria, such as *Streptococcus thermophilus*, and bifidobacteria have been shown to inhibit a broad range of enteropathogens, including *E. coli*, *Salmonella*, *Helicobacter pylori*, *Listeria monocytogenes*, and rotavirus [19,59,60,61].

Multiple direct anti-pathogen effects have been described including inhibition of pathogens growth with the production of antimicrobial compounds, resource competition, counteracting of toxin effects, inhibition of virulence, anti-adhesive and anti-invasive effects, and competitive exclusion by competition for binding sites or stimulation of epithelial barrier function [19,62]. Probiotic strains of *Lactobacillus* species have also been shown to reduce the biofilm formation in pathogenic bacteria, such as *Listeria monocytogenes*, through competition, exclusion, and displacement [63].

Competition for binding sites on host cells is common between lactobacilli/bifidobacterial and some enteropathogens, since they share carbohydrate-binding specificities. Steric hindrance at the intestinal level is an anti-attachment mechanism described in probiotic strains against pathogenic bacteria [19,59].

Probiotics can produce a wide range of antimicrobial substances, including lactic and acetic acids [19,39,64], ethanol [61], bacteriocins [62,63], and other antimicrobial compounds, such as reuterin [61].

Probiotics also have a role against viral pathogens [39,65]. It has been shown that *Bifidobacterium breve* and different *Lactobacillus* species can inhibit the absorption of the virus to the intestinal cells [39], mainly by steric hindrance or fortifying the mucosal epithelial barrier [39] or by competition for viral receptors on enterocytes [39,66]. Probiotics have also anti-fungal properties, for example *Lactobacillus reuteri* against *Candida* growth [61,67].

### 2.3 Other Activities

Other related activities have been reported in different probiotic strains, including antioxidant activity, anticarcinogenic properties, inhibition of α-glucosidase, or cholesterol lowering effects [46,62,68], due to the potential of probiotics to biosynthesize health-promoting compounds, such as vitamins (B vitamins), gamma-aminobutyric acid (GABA), bioactive peptides, or conjugated linoleic acid [15].

## 3. Safety Issues Regarding the Use of Live Probiotics

Despite their widespread use and the large body of evidence supporting the use of probiotic supplementation in different conditions, several concerns have been raised about the possibility of adverse events associated with the use of live strains, particularly in the pediatric populations and in adults with underlying diseases [32,36,69,70].

One of the main concerns about the use of live probiotics is that live bacteria may translocate from the intestine to the locally-draining tissues and blood, thereby causing bacteremia, particularly in immunocompromised, critically-ill subjects and in the pediatric populations [32,52].

Other concerns with the use of live probiotics are the possible acquisition/transmission of antibiotic resistance genes by the probiotic strains via horizontal gene transfer in the human digestive tract [14,71,72], the presence of deleterious metabolic activities, and the excessive immune stimulation in susceptible individuals [69]. Strict assessment of the probiotic strains before marketing of the product should be performed, including genome strain characterization, to assure, among others, the absence of resistance determinants [73]. A recent study has shown the ability of food-borne *Lactobacillus* in diffusing their antibiotic resistance traits to food pathogens under in vitro and in vivo conditions, thus raising concern of their use as probiotics or food supplements [74].

In the case of neonates, there is also concern that live probiotic strains may form a persistent colony that could prevent normal colonization with other microbiota or with the normal core microbiome in the GI tract, with subsequent alteration of normal immune system development [32,75]. In this regard, a combination of probiotic strains instead of a single strain has been proposed in neonates, taking into account the complexity of gut microbiome and the pathogenesis of certain diseases in preterm infants, such as necrotizing enterocolitis (NEC) [76]. The use of heat-killed probiotics (*S. thermophilus*) in enteral formula in pre-term infants has also been proposed to avoid interference with gut colonization [77].

While adverse events associated with the use of live probiotics are mainly described in case reports, in the design of randomized controlled trials key safety parameters often lack, as concluded in a recent review of 384 randomized controlled trials assessing probiotics, prebiotics, or symbiotics, recommending that an evaluation of the benefit–risk balance should always be included [24].

This benefit–risk balance is particularly important in vulnerable patients, as concluded in a systematic review of randomized controlled trials in which probiotics were used for the prevention of *Clostridium difficile*-associated diarrhea (CDAD) in adults and children. The short-term administration of probiotics appeared to be safe and effective in combination with antibiotics in patients who were not immunocompromised or severely debilitated, thus concluding that vulnerable patients should be informed of the potential benefits and risks of probiotics [23].

One important concern of safety of probiotic products is the risk of translocation and the subsequent bacteremia and sepsis. Some strains have good adherence properties on the intestinal mucosa, a mechanism associated with higher probability of bacterial translocation from gut to blood and other tissues, particularly in patients with epithelial barrier dysfunction [11,68]. This risk is of particular concern in neonates, particularly in critically ill and/or extremely preterm neonates with potentially compromised gut integrity, as described in case reports [14,26,78,79], and animal models in which the presence of immune deficiency in neonates may put them at particularly high risk of probiotic sepsis [11,80].

Although none of the randomized clinical trials have reported probiotic sepsis, there are case reports of serious infections such as septicemia, pneumonia, meningitis, endocarditis, and abscess in patients treated with different probiotics, including *Lactobacillus*, *Bifidobacterium*, *Bacillus*, and *Streptococcus*, particularly in children and adults with underlying diseases [14,69].

Since bacteremia due to probiotics usually occurs in intensive care settings, hand hygiene is recommended when manipulating central venous catheters and handling probiotic preparations [69].

In this regard, the most common adverse event associated with probiotics is fungemia in patients treated with yeast preparations (containing *Saccharomyces cerevisiae*/*Saccharomyces cerevisiae boulardii*), particularly in critically ill patients, with severe systemic gastrointestinal disease or immunosuppressed [70,81,82].

Recently, two case reports of fungemia after probiotic treatment with yeast probiotics have been published [70,83].

An eight-year-old patient in a pediatric surgical intensive care unit developed *S. cerevisiae* fungemia following treatment containing the yeast [83] and a case of fungemia due to *Saccharomyces cerevisiae* var. *boulardii* has been reported in an immunocompromised 73-year-old patient on chemotherapy and on treatment with a probiotic product (Floratil^®^, containing 0.5 × 10^9^ cells of *Saccharomyces cerevisiae* var. *boulardii*/capsule) for the management of antibiotic-associated pseudomembranous colitis [70]. Translocation of the yeast from the gastrointestinal tract to the blood was proposed as the most likely mechanism [70].

Based on this information, safety issues with the use of live probiotics, including yeasts, should always be in mind in the clinical practice, particularly in neonates, and critically ill or immunosuppressed patients [39,51], and clinical guidelines should also include safety considerations. Published safety data regarding the broad range of probiotic strains added to food or feed in food are periodically compiled by the European Food Safety Authority (EFSA) (EFSA Scientific Opinion, 2016).

These concerns prompt consideration of alternative agents such as prebiotics, postbiotics (products of microbial fermentation), specific components of probiotic strains [32], and heat-killed probiotic strains [52].

## 4. Characteristics of Heat-Killed Bacteria with Health Benefits, Including Tyndallized Bacteria

Inactivation of probiotics can be achieved by different methods, including heat, chemicals (e.g., formalin), gamma or ultraviolet rays, and sonication, with heat treatment being the method of choice for inactivation of probiotic strains in most cases [14,27,84].

Different methods of inactivation may affect structural components of the cell differently, and influence their biological activities [14,27].

As reviewed in this article, after heat treatment, industrially-grown probiotic bacteria, including bacterial extracts and supernatants in most cases, maintain their main probiotic properties at the intestinal level, thus allowing the development of safer preparations with more optimal pharmaceutical properties (long shelf-lives, etc.) [27,59,85].

Heat treatments of bacterial suspensions can use a range of temperatures between 70 and 100 °C and in some cases, inactivation is obtained with the combination of heat treatments with incubation periods at lower temperatures (ambient temperatures, cooling or freezing temperatures), a process known as tyndallization, due to the similarities with the method of sterilization to remove spores based on repeating boiling and incubation, developed by the physicist Dr John Tyndall during the nineteenth century [86,87].

**Figure 2:**
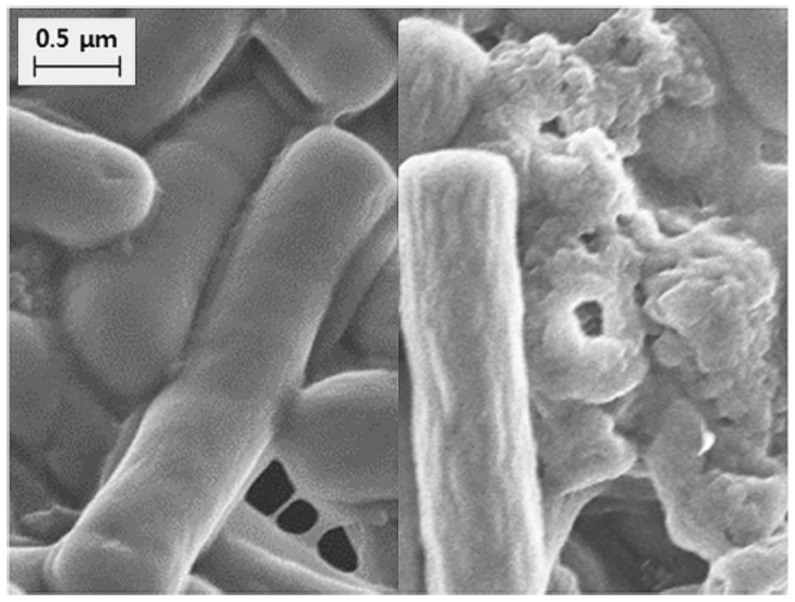
Comparison with non-treated (left panel) and tyndallized *L. rhamnosus* (right panel) [85].

A modified tyndallization process has been used to produce heat-treated industrially-grown bacteria for different uses [30,85,86]. In most of cases, the tyndallized product contains cell fractions and supernatants [85], thus taking profit of both cell structures and excreted bacterial factors. Research studies are necessary to assess the influence of the tyndallization process on the bacterial cells, since the cell structure and cell components can be disrupted/graded to different extents. In *L. rhamnosus* strains, it has been reported that the tyndallization process altered the cell form, with the presence of shrunk and fragmented cells (Figure 2) [85]. Moreover, tyndallization and other heat-treatments can lead to rupture of cell walls, with the release of cytoplasmic contents (bacterial lysates), such as DNA; and cell wall components, such as peptidoglycans, lipoteichoic acids, or heat labile pili. The released bacterial components play key immunomodulating roles [27] and can also have a role in the inhibition of pathogens [28,29].

To date; however, there is limited research on the effects that different types of inactivation treatments have on bacterial structure and components and on maintenance of probiotic properties, both qualitatively and quantitatively [27,88].

At the clinical level, there is currently increasing interest in the use of heat-killed preparations of different probiotic strains, from lactic acid bacteria and *Bifidobacterium*, in the management of a variety of diseases [84], mainly intestinal [30], but also for other diseases, for example, as support in *Helicobacter* therapy [59,89], allergic respiratory diseases [90], or topical diseases [85].

To date; however, the use of products containing heat-killed bacteria with health benefits is not completely widespread. Medical devices containing different tyndallized strains in combination with mucosal protectors, such as xyloglucan or gelatin tannate, are being recently marketed for the treatment of colic in children and adults (for example, xyloglucan plus tyndallized *L. reuteri* and *B. breve* strains) and for the treatment of diarrhea and for the prevention of gut dysbiosis associated to diarrhea (gelatin tannate plus tyndallized *Lactobacillus acidophilus*, *Lactobacillus plantarum*, *Lactobacillus casei*, *Lactobacillus rhamnosus*, *Bifidobacterium bifidum* and *Streptococcus thermophilus*). In these products, synergism between mucosal protectors and probiotic strains are sought in terms of immunomodulation, cell barrier integrity, and competition against pathogens. Tyndallized *Lactobacillus acidophilus* HA122 (2 × 10^9^ CFU/2 mL), in combination with extracts of *Matricaria chamomilla* and *Melissa officinalis*, is also marketed for the treatment of infantile colic.

## 5. Bacterial Cell Lysis as a Pre-Requisite for the Physiological Effects of Probiotics

Contrary to what is commonly believed, bacterial viability or bacterial cell wall integrity is not an essential condition for the intestinal effects of probiotics, as reviewed in the next section of this manuscript. In fact, key molecules from gut bacteria, including LPS or peptidoglycan, interact with eukaryotic receptors when they are released into the environment from disrupted or completely-lysed cells or during the bacterial growth process [91,92,93]. It has been recently shown that the degradation and lysis of bacteria by lysozyme enhance the release of bacterial products, including peptidoglycan, that activate pattern recognition receptors in host cells, this being the process important for the resolution of inflammation at mucosal sites [93].

**Figure 3:**
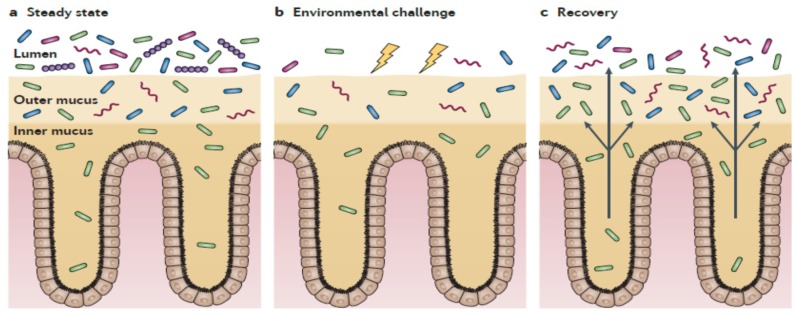
Gut biogeography of the bacterial microbiota [94]. (**a**) A subset of species (green) is able to penetrate the inner mucus layer and enter crypt spaces. (**b**) Environmental challenges such as diet perturbations, antibiotic consumption or abnormalities in gastrointestinal motility massively alter the lumen community. However, the more stable mucosal environment and the crypts protect important bacterial species. (**c**) The crypts and mucosa serve as reservoirs to repopulate the lumen.

This is also supported by the localization of gut microbiota in the colon in the absence of mucosal damage, mainly present in the outer mucus layer, which offer nutrients, and distanced from enterocytes by a firmer inner structure, which is almost devoid of bacteria and confers protection to the host [94] (Figure 3). Only certain types of bacteria, for example, Proteobacteria (including enterobacteria), are able to penetrate the mucus layers and reside in close proximity to the host cells [94]. Therefore, in this scenario, one can speculate that the probiotic effects, exerted by both gut microbiota in normal conditions or by probiotics taken from supplements, are mainly derived from the release of bacterial products, which can pass through the mucus and stimulate the epithelial cells more directly than whole cells can [68]. Therefore, in comparison with live bacteria, the use of heat-killed bacteria, providing disrupted cells and released bacterial components, could better reproduce in vivo the physiological conditions in the gut lumen and outer mucus layer, with key components reaching eukaryotic cells and enhancing the mucosal integrity.

Additionally, taking into account the gut biogeography of gut microbiota, with three defined levels (lumen, outer mucus, and inner mucus) (Figure 3), the passage of active components from heat-killed probiotics to reach the epithelium seems to be a gradual process, where not all molecules would reach the eukaryotic receptors in vivo. Therefore, one can speculate that the benefits would concentrate in the apical side of the mucosa, maintaining its integrity. Moreover, the immunomodulatory properties of probiotics observed in in vitro models using different immune cells would probably be reduced in the human intestine in vivo, in which the mucus layers in the colon create a boundary between the gut lumen and the host tissue [94].

## 6. Effects of Probiotics as Heat-Killed Bacteria

Different strains, including lactic acid bacteria and bifidobacteria, are able to produce beneficial effects in their heat-inactivated form [14]. There is also considerable data showing that not only dead cells, but also metabolites, cell fractions, and culture supernatants of probiotic bacteria can exert biological effects [22,27,32,95]. The use of them is based on the evidence suggesting that individual effector molecules interacting with host cells may underlie probiotic effects [35,96,97]. Although similar benefits can be obtained with the different strategies—live, heat-inactivated, or different fractions [98]—relevant differences could exist among all of them [27].

For example, while live probiotics can have difficulties in attaching to intestinal epithelial cells to modulate immune responses, due to the mucous layer that avoids direct contact between bacteria and epithelial cells, microbial products can pass through the mucus and stimulate epithelial cells more directly [68]. In any case, the mechanisms by which non-viable bacteria and different bacteria fractions can exert their effects need further research.

Various microbiological components, such as cell-free supernatants [68], exopolysaccharides (EPS) [99], teichoic and lipoteichoic acids [35,100], peptidoglycans, LPS [91], and metabolites (De Marco et al., 2018) have anti-inflammatory and immunomodulating activities, through stimulating the innate immune system (Adams, 2010), the adaptive responses [101] and through their effect on the integrity of the intestinal mucous membrane [19,35]. Heat-killed probiotics are also able to antagonize pathogens (with antimicrobial compounds and by competition with pathogens for adhesion and colonization) [35,99].

These specific components are usually active on Toll-like and other signal transduction receptors in the intestinal epithelium, dendritic cells, and other immune intestinal cells [32].

In this section we review the immunomodulating effects and competition activities against pathogens of both heat-killed preparations of beneficial bacteria and purified cell-wall components, such as lipoteichoic acids, peptidoglycan, or EPS. The main effects of heat-killed probiotics and supernatant fractions are summarized in Table 2 and Table 3.

### 6.1. Immunomodulating Effects of Heat-Killed Probiotics and Purified Components

#### 6.1.1. Heat-Killed Bacteria

A body of evidence indicates that inactivated bacteria have immunomodulatory effects, which can be similar to that observed with live bacteria [27]. Interestingly, inactivation, with the subsequent loss of viability and cell lysis, can produce further and more complex immunomodulation than expected [27].

##### Lactic Acid Bacteria

Lactic acid bacteria can modulate immune responses, with the induction of IL-12 secretion that enhance the innate immunity [38].

In a recent study in mice, immune responses induced by different heat-killed *Lactobacillus* species were compared, indicating that *L. paracasei* had the highest capacity to induce IL-12 secretion in comparison with other *Lactobacillus* species, including *L. reuteri*, *L. casei*, and *L. plantarum* [38].

Combination of heat-killed multispecies of lactic acid bacteria have also been tested (including *L. acidophilus*, *L. plantarum*, *L. fermentum*, and *Enterococcus faecium*). Enhanced immunomodulatory activity in mouse macrophages was reported in comparison with the same combination containing live strains [102]. Heat-treatment at 100 °C for 30 min did not alter the capacity of these strains to adhere to Caco-2 cells, while treatment at 121 °C for 15 min reduced more than 50% of their adherent capacity [102].

Heat-killed probiotic strains also maintain their capacity to induce secretory IgA production, as demonstrated in fecal samples from pre-term infants treated with a formulation containing heat-killed *S. thermophilus* [77].

Heat-killed probiotic bacteria have also been shown to have an effect in the maintenance of barrier integrity. For example, heat-killed *L. rhamnosus*, strain OLL2838, has been shown to protect against mucosal barrier permeability defects in mice with induced colitis [103]. In Caco-2/TC7 cell monolayers infected with diarrheagenic, diffusely adhering Afa/Dr *E. coli* C1845, heat-killed *L. acidophilus* LB plus its culture medium counteracted *E. coli*-induced increase in paracellular permeability [104].

In a study in rats with acute alcohol intestinal injury, the administration of heat-killed bacteria of the probiotic product VSL≠3, containing *B. breve*, *B. longum*, *B. infantis*, *L. acidophilus*, *L. plantarum*, *L. paracasei*, *L. bulgaricus*, and *S. thermophilus*, significantly protected the cyto-architecture of the intestinal barrier, preventing passage of endotoxin and other bacterial products from the gut lumen into the portal circulation and down-regulating the expression of TNF-α [105].

##### Bifidobacterium

In a comparison between live and heat-killed *B. breve* M-16-V, both forms showed immunomodulating effects that suppressed pro-inflammatory cytokine production [106].

Heat inactivated *B. bifidum* OLB6378 can also act on sIgA production, as observed in a mouse intestinal explant model, being the result of a direct microbial effect on the intestinal epithelium [107].

#### 6.1.2. Cell Wall Components

Currently, there is increasing interest to understand the biological activities of cell wall components of probiotic bacteria in the design of new advanced therapeutics and to avoid the use of live microorganisms [28]. In the case of the development of products containing heat-killed strains, the identification of key cell wall components is also necessary, together with the assurance that these molecules maintain their activity after the heat treatment.

Despite their biological importance, cell wall components of probiotics are poorly characterized [120]. Peptidoglycan and lipoteichoic acids are the major cell wall components of Gram-positive bacteria and can be considered the pivotal components for the immunomodulating effects of most probiotics [35,97,120]. While lipoteichoic acids and peptidoglycan from *Lactobacillus* species have been associated with immunomodulating effects in different models [35,41,121], in the case of bifidobacteria, the immunomodulating roles of these molecules have not yet been properly studied [28].

##### Lipoteichoic Acids

The role of lipoteichoic acids as IL-12 inducers, thus activating the innate immune functions, have been demonstrated in *L. plantarum* in cultures of mouse spleen cells and splenic dendritic cells [121]. Lipoteichoic acid from *L. plantarum* also confers anti-inflammatory responses, as observed in a study in porcine intestinal epithelial cell lines. Of note, lipoteichoic acids, suppressed poly I:C-induced IL-8 production, suggesting the capacity of these molecules to inhibit viral pathogen-induced inflammatory responses in intestinal epithelial models [35].

##### Peptidoglycans

Peptidoglycan from *L. rhamnosus* has been shown to improve innate immune responses in immunocompromised-malnourished mice after *Streptococcus pneumoniae* infection. Moreover, nasal administration of this molecule improved innate immune responses and induced respiratory and systemic adaptive human responses [122]. Peptidoglycans from different *Lactobacillus* species have also the capacity to inhibit the release of inflammatory cytokines in models of LPS-induced macrophage-like cells [123].

#### 6.1.3. Exopolysaccharides and Surface-Layer Proteins

##### Exopolysaccharides

Exopolysaccharides (EPS) are secreted and extracellular surface carbohydrate polymers, which can be loosely attached to the bacterial cell surface or released into the surrounding cell environment [29,124]. Present in most bacteria, they act as a protective surface layer, and also interact with the surrounding environment [29], mainly in bacterial biofilm formation, in which the EPS can be produced within individual bacterial strains and also by different species [125].

A wide variety of EPS functions have been characterized in probiotic bacteria, including immunomodulating and pathogen protection properties [29,124]. Due to their biological functions and physicochemical properties, bacterial EPS are being extensively studied due to their potential applications at the industrial, food, cosmetic, or medical levels [29,126]. A growing number of studies are reporting in vivo and in vitro immunomodulating effects of EPS from strains of *Bifidobacterium* and *Lactobacillus* [29]. EPS has been suggested to be involved in the cross-talk between probiotic bacteria and host immune system, potentially playing a role in intestinal homeostasis via interaction with intestinal epithelial cells [28,127].

In the case of *B. breve*, the immunomodulating role of EPS has been demonstrated by comparison between EPS-positive and EPS-deficient strains [29,124]. In *Lactobacillus* species, different EPS have exhibited immunomodulatory effects in cultures of immune cells, while only limited studies have reported their interaction with intestinal epithelial cells [127].

##### Surface-Layer Proteins

Surface-layers are paracrystalline dimensional arrays of proteins and glycoproteins that overlay the cell surface of several genus and species of Bacteria and Archaea, forming a symmetric, porous layer that completely covers the cell surface [128].

S-layer proteins are present on the cell surface of some lactobacilli. For example, S-layer protein A from *L. acidophilus*, has been associated with the ability of the probiotic to bind to dendritic cells to induce an immunoregulatory phenotype (Treg) and to promote mucosal homeostasis [51,129].

#### 6.1.4. Cell-Free Supernatants and Soluble Factors

Cell-free supernatants contain batch culture medium, metabolites, and other secreted products that can cross the mucus layer and reach the intestinal monolayer of epithelial cells and interact with mucosal immune cells [95,110]. Probiotic metabolites have anti-inflammatory and antioxidant activity, acting first on intestinal epithelial cells and then on immune cells, with differences depending on the probiotic strain [68]. Reduction of the production of pro-inflammatory mediators have been demonstrated in in vitro models of immune cells upon exposure to secreted products from *Lactobacillus* [68,108] and *Bifidobacterium* species [68,110].

In a study with different probiotic strains (including *L. delbrueckii*, *L. paracasei*, *L. salivarius*, *L. reuteri*, *L. rhamnosus*, *L. acidophilus*, *L. plantarum*, *L. lactis*, *L. casei*, *S. thermophilus*, *B. breve*, and *B. longum*) in peripheral blood mononuclear cells (PBMC), the anti-inflammatory immune responses observed were mediated by both metabolites and cell-surfaces of these bacteria [130]. In models of colon epithelial cells, soluble purified peptides secreted by *L. rhamnosus* GG have prevented cytokine-induced cell apoptosis, thus promoting intestinal epithelial homeostasis [19,109], and cell-free supernatants of *L. acidophilus*, *L. casei*, and *L. reuteri*, containing metabolites, were able to downregulate the expression of PGE-2 and IL-8 [68].

Identification of the key metabolites with immunomodulating effects present in cell-free supernatants would deserve further research.

### 6.2. Protective Effects against Pathogens of Heat-Killed Probiotics and Purified Components

Protection against pathogens, by the production of substances (metabolites and bacteriocins), preventing pathogens adhesion and invasion, and also preventing biofilm formation by pathogenic bacteria, has also been described in heat-killed bacteria, in cell-free supernatants, and in purified compounds, particularly EPS [29,99], thus supporting their use as an alternative strategy to live probiotics.

#### 6.2.1. Heat-Killed Probiotics

Competition for adhesion sites at th gastrointestinal level has been described between heat-killed cells/purified structures from *Lactobacillus* and gastrointestinal pathogens, such as diarrheagenic *E. coli* (ETEC) [111], *Campylobacter* [112], and *H. pylori* [59,89].

In a mice model of *Salmonella* infection, the combination of heat-killed multispecies of lactic acid bacteria (including *L. acidophilus*, *L. plantarum*, *L. fermentum*, and *Enterococcus faecium*) was able to reduce *Salmonella* invasion and the induced inflammation [102], this being the effect attributed to lipoteichoic acids and EPS [102]. Heat-killed *L. plantarum* also protected against *Salmonella* infection in mice and reduced translocation of this pathogen into different organs, such as spleen or liver, mainly by inhibiting pathogen adhesion and invasion [113].

Heat-killed lactobacilli has also exhibited activity against *H. pylori*. In vitro, heat-killed *Lactobacillus johnsonii* inhibited the growth of *H. pylori*. Moreover, the number of *H. pylori* in the infected stomach of germ-free mice was significantly decreased by the repeated oral administration of the heat-killed strain, with deformations in *H. pylori* cells being observed (disappearance of spiral, bending of cell body, coccoid formation, degradations, etc.) [89].

The oral administration of inactivated bifidobacteria also led to an enhanced resistance of mice to *Salmonella* infection [114]. In an in vitro study, heat-inactivated *Bifidobacterium* BB12 interfered with the formation of *Streptococcus mutans* biofilms in dentinal cavities [115].

#### 6.2.2. Cell Wall Components

##### Cell Wall Polysaccharides

Complexes of polysaccharide-peptidoglycan from *L. casei* strain YIT9018 have been shown to have anti-infectious activities against *L. monocytogenes* and *P. aeruginosa* [120,131].

#### 6.2.3. Exopolysaccharides and Surface-Layer Proteins

##### EPS

Protection against pathogens has been described in purified EPS from lactic acid bacteria and bifidobacteria [28,29], through their anti-adhesive properties against pathogens (mainly enterobacteria) and also through the stimulation of the immune response against pathogens. EPS has also been shown to decrease the cytotoxic effects of bacterial toxins in Caco-2 cells [132]. In fact, some authors postulate that these protective actions of EPS-producing probiotics could be related to the formation of a protective film, preserving the host cells against injury, for example, by pathogens or their toxins [29]. Moreover, EPS from bifidobacteria has been shown to facilitate the growth of lactobacilli along with other anaerobic bacteria [28].

Bifidobacteria strains are popularly associated with EPS, with high structural diversity among strains. EPS form an interfacial layer separating the bacteria from its surrounding environment, considerably contributing to their anti-pathogenic activity [28]. In animal studies, the administration of *B. breve*, producing EPS, reduced colonization of *Citrobacter rodentium*, in comparison with the mutant strain [124,133]. EPS isolated from *B. bifidum* facilitated the growth of lactobacilli and other anaerobic bacteria and inhibited the growth of enterobacteria, enterococci, and *Bacteroides fragilis*. EPS from *B. longum* also inhibited pathogenic bacteria growth, including *E. coli*, *Salmonella*, *S. aureus*, *B. subtilis*, and *B. cereus* [134].

*S. thermophilus* CRL1190 strain reduced *H. pylori* adhesion and attenuated inflammatory response in AGS cells, being the first demonstration of the capacity of this strain to adhere to the stomach gastric mucosa, and to improve protection against *H. pylori*, being these effects attributed to the EPS [135]. These characteristics convert different EPS in promising candidates in developing functional food and medical devices for the management of different diseases [29,99].

The antagonistic effect of isolated EPS from lactobacilli has also been assessed in in vitro and in vivo studies [29]. Purified EPS from *L. plantarum* WLPL04, consisting of xylose, glucose and galactose, was able to inhibit the adhesion of *E. coli* O157:H7 to HT-29 cells in competition, replacement, and inhibition assays. Additionally, the EPS exhibited strong inhibition against biofilm formation by pathogenic bacteria, including *Pseudomonas aeruginosa*, *E. coli* O157:H7, *Salmonella*, and *Staphylococcus aureus* [99].

In fact, EPS molecules from probiotics would have structural and biological similarities to other non-bacterial polymers, for example, xyloglucan, a vegetal polymer (from the seeds of *Tamarindus indica*) contained in different medical devices and currently used in the management of different gastrointestinal diseases [4]. Since xyloglucan also has protective film-forming properties against *E. coli* or *Salmonella* [4], synergism can exist with heat-killed probiotics, thus supporting their combined use in gastrointestinal diseases, as is the case of medical devices containing heat-killed probiotics and mucosal protectors (xyloglucan and also gelatin tannate).

The difficulties in the purification of EPS from bacterial cells support the use of other polymers with similar properties, for example xyloglucan [4].

##### S-Layer Proteins

Although poorly understood, protective properties against pathogens have been described in the case of *Lactobacillus* S-layer proteins [128]. Surface-layer protein extracts from *Lactobacillus helveticus*, strain R0052, has prevented EHEC O157:H7 binding to epithelial cells in vitro [51,136]. Exposure of epithelial cells with S-layer protein extracts decreased *E. coli* O157:H7 adherence and attaching-effacing lesions and preserved the epithelial barrier function [136].

#### 6.2.4. Cell-Free Supernatants

Cell-free supernatants from probiotic bacteria contain a wide range of compounds with anti-microbial properties, including organic acids, such as lactic acid, hydrogen peroxide, diacetyl, reuterin, and bacteriocins [137,138].

The production of organic acids by multiple probiotic strains, belonging both to lactic acid bacteria and bifidobacteria, is mainly responsible for the antimicrobial activity against Gram-negative pathogens [138]. Exposure of *C. difficile* to filtered supernatants from *S. thermophilus* has shown a dose-dependent, bactericidal effect due to lactic acid [139].

Reuterin (3-hydroxypropionaldehyde) is a well-known antimicrobial metabolite produced by *L. reuteri*, and thought to exert its effect by oxidizing thiol groups in the target gut pathogenic microorganisms [138,140].

##### Secreted Bacteriocins

Bacteriocins are antibacterial small heat-stable peptides that are able to inhibit the growth of other bacteria, including enteric pathogens [39,51], (Bactibase Database http://bactibase.hammamilab.org/main.php). Exceptionally, few bacteriocins, together with their native antibacterial property, also exhibit additional anti-viral and anti-fungal properties. Bacteriocins from Gram-positive bacteria, especially from lactic acid bacteria, have been thoroughly investigated considering their great biosafety and broad industrial applications [116].

Inhibition of the in vitro growth of a broad range of pathogens, including *Clostridium*, *Bacillus*, *Listeria*, *Enterococcus* and *Staphylococcus*, enterobacteria, and other Gram-negative bacteria and in vivo protection against infection has been described in different lactic acid bacteria [39,116,117].

Bifidobacteria release a wide diversity of bacteriocins, being considered the main factor responsible for the antimicrobial activity of the cell-free supernatants [28]. Bifidocins, isolated from different *Bifidobacterium* strains, have exhibited a wide range of bactericidal activity, against Gram-positive and Gram-negative bacteria and some yeasts, through cell lysis. Another bacteriocin produced by *Bifidobacterium*, acidocin, has been shown to inhibit *Clostridium* species in fermented food products [28,118,119].

Bacteriocins and other antimicrobial compounds can be present in the heat-inactivated probiotic products, since they can resist temperatures up to 100 °C [116]. Other interesting properties of bacteriocins to be considered good candidates as possible ingredients in new-generation probiotic products are their stability in a wide pH range of 3–10, and towards the action of weak organic solvents, refrigeration, freezing, and action of salts and enzymes [28].

Nevertheless, the presence and activity of antimicrobial compounds in products containing heat-inactivated bacteria and their culture medium deserve further research.

## 7. Protective Barrier Properties of Tyndallized Probiotics in Combination with Mucosal Protectors in Intestinal In Vitro Models

One common property among the different strains of probiotics is their capacity to fortify the intestinal mucosal barrier [19,48]. These effects have also been observed in heat-inactivated probiotics [103] and in purified components, such as EPS (Castro-Bravo et al., 2018), and also in the group of components with mucosal protective properties such as xyloglucan and gelatin tannate [4]. Synergism between tyndallized probiotic strains and mucosal protectors have been demonstrated in in vitro models of intestinal cells.

In in vitro models of intestinal mucosa (HT29-MTX cells), the combination of tyndallized strains, including *L. acidophilus*, *L. plantarum*, *L. casei*, *L. rhamnosus*, *B. bifidum*, and *S. thermophilus*, and gelatin tannate protected intestinal cells from *E. coli* infection by inhibiting the adhesion and internalization of bacteria, preventing the increase of paracellular permeability and modulating cytokine gene expression [52,141].

The same combination was also assessed in *E. coli*-infected CacoGoblet^®^ cells, with an increase in the transepithelial electrical resistance (TEER) and a reduction in the paracellular flux, being these effects more important than those observed with the heat-killed probiotic mixture alone, *S. boulardii* or the anti-diarrheal agent diosmectite. These results highlight the synergism between a mucosal protector and heat-killed probiotics to protect the intestinal barrier integrity and to prevent enteropathogens adhesion and invasion. Synergism has also been proposed in terms of onset of action, in which the presence of the mucosal protector would produce a faster onset of action of the probiotic mixture [142].

The protective properties of tyndallized probiotics plus other mucosal protectors, such as xyloglucan, should deserve further research, in intestinal cells and also in other models, as nasal epithelial cells, based on the previous studies supporting the use of xyloglucan as protector of the nasal mucosal epithelial cells [143,144]. In fact, xyloglucan in nasal formulations is an innovative strategy for the management of nasal disorders, as rhinitis and rhinosinusitis, based on their protective properties on the nasal epithelial cells, maintaining the barrier integrity and allowing the avoidance of allergens and triggering factors, as demonstrated in MucilAir™Nasal cells [4,143,144] and in patients with rhinosinusitis [145].

Although experience with probiotics for the treatment of nasal disorders is limited, recent data from patient biopsy specimens also indicate that topical heat-killed probiotics can be a safe and feasible alternative treatment, through their anti-inflammatory properties [90]. Further research; however, is needed to assess the clinical effects of heat-killed bacteria in nasal disorders and also in combination with mucosal protectors such as xyloglucan.

## 8. Clinical Benefits of Tyndallized Bacteria as Probiotics in Gastrointestinal Diseases

### 8.1. Bloating

In a recent double-blind, multicenter, randomized clinical trial in adult subjects with a diagnosis of functional bloating, the administration of a medical device containing the mucosal protector xyloglucan plus tyndallized *L. reuteri* and *B. breve*, during 20 consecutive days, produced higher symptoms relief than simethicone, particularly regarding abdominal distension and flatulence. Of note, at baseline, all subjects had a diagnosis of small intestinal bacterial overgrowth (SIBO) confirmed by the hydrogen breath test, while at the end of treatment a reduction in hydrogen gas production was observed in both treatment arms [31].

SIBO is a common gastrointestinal dysbiosis that can be caused by the overuse of certain drugs such as proton pump inhibitors. The long-term reduction of gastric secretion creates favorable conditions for the colonization of various bacterial species in the upper gastrointestinal tract [146]. Moreover, it is also known that *H. pylori* infection can also alter the microbiota of the upper gastrointestinal tract, and active *H. pylori* infection has been found to be significantly associated with the presence of SIBO [146,147].

SIBO is due to the overgrowth of species that commonly colonize the colon, mainly Gram-negative, strict anaerobes, and Enterococci [148]. Interestingly, in children with SIBO, higher counts of *Salmonella* have been detected in fecal samples, leading to the assumption that individuals with SIBO possibly have dysbiosis in different intestinal segments and not only in the small intestine [149].

In this context, we can speculate that, while the effect of simethicone on SIBO is through its de-foaming properties, altering the elasticity of interfaces of mucus-embedded bubbles in the gastrointestinal tract [150], the effect of the medical device in reducing SIBO and the associated symptoms would be more associated with the protective effects against pathogens produced by the probiotic strains and the mucoadhesive properties of xyloglucan, with antiadhesive properties against enterobacteria, as already demonstrated in in vitro [151] and in vivo studies [152].

### 8.2. Pediatric Disorders

With the use of new sequencing techniques, gut microbiota and the characteristics of dysbiosis is currently being assessed in detail in pediatric populations, particularly in infants and preterm infants. Recent findings suggest that the immature intestinal mucosa and gut dysbiosis in infants precedes the development of relevant severe diseases, as late-onset sepsis [153] or necrotizing enterocolitis (NEC) [54], and also as less severe, but particularly stressful for parents, infantile colic [87]. It has been recently shown that at weaning the intestinal microbiota induces a vigorous immune response (the “weaning reaction”) that is programmed in time, and inhibition of this effect leads to pathological imprinting and increased susceptibility to colitis, allergic inflammation, and cancer later in life [154].

*B. breve* is the dominant species in the gut of breast-fed infants and it has also been isolated from human milk. For this reason, strains of *B. breve* are widely used in pediatrics, having antimicrobial activity against enteropathogens and immunomodulatory effects. Of note, it is devoid of transmissible antibiotic resistance traits and cytotoxicity [133].

Probiotic supplementation with strains of *L. reuteri*, originally cultured from mother´s breast milk, endowed with immunomodulating effects, have been shown to reduce the incidence and severity of severe infant diseases, such as NEC [54] or late-onset sepsis [155], and have also been tested in infantile colic [87].

Particularly in neonates; however, it is important not to alter the gut bacterial colonization [77], thus supporting the use of heat-killed strains, for example in neonates in enteral nutrition [77] or for the management of infantile colic [30,87,156].

#### Infantile Colic

Infantile colic is a common condition (20% of infants) occurring during the first four months of life, defined as infant irritability, fussing, or crying that occur without obvious cause, without evidence of infant failure to thrive, fever, or ill health, presenting with recurrent prolonged periods [87,156]. To date, infantile colic pathophysiology is poorly understood, with the presence of gut microbiota dysbiosis, barrier alterations, and mild chronic gastro-intestinal inflammation [87]. Gut dysbiosis in colicky infants is characterized by decreased levels of bifidobacteria, lactobacilli, and butyrate-producing species and increased levels of Proteobacteria, leading to a more pro-inflammatory environment [87]. Moreover, intestinal mucosal immaturity has also been reported, with the possible entry of toxic compounds from the gut lumen to the blood [87,157].

This knowledge and the dissatisfaction with conventional treatment options (for example, simethicone) is opening new therapeutic strategies for the management of the disease, particularly based on the use of probiotic heat-killed strains [87,156].

In a recent pilot study in 46 infants aged three to 16 weeks with infantile colic, the administration of xyloglucan plus tyndallized *Lactobacillus reuteri* SGL01 and *Bifidobacterium breve* SGB01, at 100 × 10^9^ CFU/g, significantly decreased the mean duration of crying episodes, in comparison with a lactase dietary supplement. These results suggest a role of the combined use of xyloglucan plus tyndallized bacteria in the management of infantile colic, although further research in larger studies is needed [30].

Administration of tyndallized *Lactobacillus acidophilus* HA122 (2 × 10^9^ CFU/2 mL), in combination with extracts of *Matricaria chamomilla* and *Melissa officinalis* produced a significant reduction of the mean daily crying time in comparison with simethicone, in a recent randomized open-label controlled clinical trial in children aged between two weeks and four months old [156].

Based on these results, the use of tyndallized bacteria in combination with mucosal protectors could also be considered in the prevention strategies of the disease.

### 8.3. Diarrhea

Heat-killed *L. acidophilus* LB has been tested in adult patients with chronic diarrhea, with marked improvements in the remission of clinical symptoms at the end of treatment in comparison with live lactobacilli [158].

Heat-killed bacteria have also been tested in children with diarrhea. Lyophilized, heat-killed *L. acidophilus* LB was tested vs. placebo in children with acute diarrhea as an adjunct to oral rehydration therapy. After 24 h of treatment, in the *L. acidophilus* LB group the number of rotavirus-positive children with watery stools was significantly lower, with a significant reduction in the mean duration of diarrhea vs. placebo [159].

In a randomized, double-blind, placebo-controlled clinical trial, in selected and controlled homogeneous groups of children with well-established, non-rotavirus diarrhea, adding lyophilized, heat-killed *L. acidophilus* LB bacteria plus their culture medium to a solution of oral rehydration solution shortened the recovery time by one day (i.e., the time until the first normal stool was passed) as compared with children who received placebo oral rehydration solution [104].

Based on the known synergism between tyndallized bacteria and mucosal protectors, such as xyloglucan or gelatin tannate, maintaining mucosal integrity and interfering with potential pathogenic bacteria [4,52], different clinical trials could be performed with the combination of different types of gastroenteritis, as for example in the prevention of antibiotic-associated diarrhea, in diarrhea in immunocompromised children, or in gastroenteritis produced by different bacterial species.

In fact, xyloglucan and gelatin tannate have already been demonstrated to reduce the main symptoms of gastroenteritis [4,160] in adults [161,162] and children [163,164].

### 8.4. Extra-Intestinal Diseases

Research on the benefits of inactivated bacteria is being extended to a variety of extra-intestinal diseases [165,166].

The use of oral probiotics is an attractive option for the management of allergic diseases, particularly atopic dermatitis [85], based on the observations that infants who develop atopic dermatitis have fewer probiotic bacteria in the gut than healthy controls [85,167] and that modification and stabilization of gut microbiota with the use of probiotics could improve gastrointestinal dysbiosis [51,85].

Results about the use of different live probiotics, mainly *L. rhamnosus* GG and *B. breve* and *B. longum*, in atopic dermatitis have generated considerable controversy in children, adults, and also during pregnancy [32,168,169], due to contrasting efficacy results and, in some cases, due to the occurrence of adverse events [32].

A body of evidence indicates that the positive effects may be related to the type of probiotic strain, the method of administration, onset time, dose, and treatment duration [35,168]. Several studies in mice have demonstrated that tyndallized *L. rhamnosus* and *L. brevis* strains can prevent the development of atopic dermatitis [170,171]. Oral administration in mice of tyndallized *L. rhamnosus* at 10^8^, 10^9^, and 10^10^ CFU/mL produced dose-dependent improvement in signs and symptoms of the disease, thus indicating their potential for the management of the disease [85]. In a recent study in a murine model of atopic dermatitis, the oral administration of metabolites from lactic acid bacteria improved skin injury [165].

In a multicenter, randomized, double-blind controlled trial, the use of a milk formula containing heat-killed *B. breve* C50 and *S. thermophilus* 065 in children at high risk of atopy reduced the incidence of digestive and respiratory potentially allergic events [172,173].

In fact, topical application of heat-killed probiotics, purified compounds, and also in combination with mucosal protectors, is receiving special attention and deserves further studies. The use of mucosal protectors in dermatological diseases is based on their protective barrier properties to avoid skin damage and their role in skin regeneration [4,174].

## 9. Concluding Remarks

Probiotics are the focus of interest at multiple levels, including consumers, patients, clinicians, scientific community, and pharmaceutical companies, and there is increasing interest to improve probiotic products, making them safer and more specific for each intended condition. In this context, new-generation probiotic products, including heat-killed strains, key components, or compounds with similar effects to living probiotic cells are being developed and already marketed, for certain indications, particularly for gastrointestinal disorders.

To date; however, there is still a number of issues to be tackled for both live probiotics and for new-generation products containing inactivated cells, cell fractions, or purified components to develop rationally-designed beneficial therapies to provide enhanced protection against infections and other diseases [17]. In general, a better understanding of the complex probiotic–pathogen interactions in the real human intestine will help to develop more specific products for each condition and to know the extent to which the bacterial-derived components are active in vivo [17,62,94,175], with a better defined benefit–risk ratio, particularly in vulnerable groups [69,70].

Currently, the use of probiotics is framed within the strategies to avoid antimicrobial resistances [4,176,177] and the need to avoid chronic pharmacological treatments and their adverse effects [4]. In fact, in the current context of high levels of antibiotic resistances, acquisition and retransfer of resistance genes should be addressed in the safety evaluation of live probiotics [71,73], and should be considered in the development of future products [73,76]. In this regard, the use of inactivated bacteria can provide important benefits, decreasing the risk of transmission of antibiotic-resistant genes.

Based on the evidence from case reports, it is clear that standard safety evaluations have to be included in randomized clinical trials assessing probiotics [24], and safety issues also have to be transmitted to health care professionals, including pharmacists in the pharmaceutical offices, where recommendation of probiotics is widespread and often obtained without medical prescription. Comorbidities and vulnerable conditions can be frequent in patients taking probiotics and; therefore, information about the possible associated risks should be given. The risk of translocation with possible systemic infections should be taken into account in vulnerable patients, and also considering certain conditions that can favor translocation, such as the presence of dysbiosis and certain conditions altering gut microbiota (for example, immunosuppression) [178].

In this regard, increasing interest is being focused on new-era products, with the use of heat-inactivated strains and purified key components responsible for the beneficial effects [28]. Purified components, such as EPS, lipoteichoic acids, metabolites, and bacteriocins, might play an important role in replacing live probiotics. In this field, more research is needed in different aspects, for example, to identify specific strains for each condition; to assess the degree of bacterial cell disruption after heat treatments (and to identify the optimal conditions that can inactivated with maintenance of the cell structure); to identify the key components of the beneficial effect for a certain strain; and to test the synergism of different combinations, which could include different heat-inactivated strains and purified key components, as well as mucosal protectors, with protective barrier properties. Moreover, the in vitro results and animal models should be interpreted considering the particular conditions of the human intestine, particularly in the colon, with a stratified layer structure where gut microbiota is mainly present in the outer layer. The physiological effect that heat-killed strains and their release compounds can exert in vivo should be also taken into account, since a substantial presence of disrupted cells or released compounds in the outer mucus layers seems to be the most probable situation, rather than a predominant direct contact with the epithelial cells.

Anyway, results reviewed in this article have shown that tyndallized bacteria clearly have favorable effects at the clinical level in the management of different diseases, representing a new generation of safer and more stable products.

As we have reviewed, the presence of key structures in the cell or supernatant fractions is able to confer probiotic properties, mainly through immune-modulation, protection against pathogens, and fortifying the mucosal barrier integrity. For the next generation products, the purification of these components and quantification of these effects would probably allow more standardization, leading to high specific and safe products intended for patient-tailored therapies. To compare and standardize these products, common activities among probiotic strains could be assessed, for example, their capacity to maintain mucosal integrity.

Current existing evidence of heat-killed bacteria in relation to health benefits indicates that they can be safe alternatives to live probiotics in vulnerable populations, such as neonates [77], and also have a role in the management of gastrointestinal disorders in children and adults, including bloating and diarrhea [30,104,156,158,159]. The synergism between tyndallized bacteria and mucosal protectors has been demonstrated in patients with bloating [30,31], while the role of this combination in other intestinal diseases and also in extra-intestinal diseases could also be explored.

This is the case of topical diseases, such as atopic dermatitis, with a demonstrated relationship to environmental pollution, and related to skin barrier dysfunction [179]. The topical use of heat-killed probiotic bacteria and mucosal protectors could provide benefits for the management of this disease, taking into account that, to date, the benefits provided by some topical protection creams are under debate [180].

Topical application of heat-killed bacteria could have also a role in the management of allergic respiratory diseases, based on the favorable results obtained with mucosal protectors in nasal in vitro models [142,144].

Another field that could be explored is in urinary tract infections (UTIs), based on the evidence indicating that mucosal protectors can reduce the intestinal reservoirs of uropathogenic *E. coli* strains [4,151,181]. The results indicating that metabolites produced by lactobacilli (hydrogen peroxide and lactic acid) act cooperatively to kill uropathogenic organisms in vitro [182,183] could be the starting point for the development of products containing heat-killed bacteria for the management of UTIs.

Preliminary in vitro data have also been obtained in *H. pylori* infection models, thus suggesting that heat-killed bacteria could also have a role in the prevention and treatment of *H. pylori* infection [89]. Although more research is needed to assess the interaction between *H. pylori* and probiotic strains and the role that probiotics (live or inactivated) can play in the prevention and in the support of antibiotic treatment strategies [184].

Overall, the reviewed data are indicating that alternatives to live probiotics, including heat-killed bacteria or their fractions or purified components, have key beneficial effects. These types of products offer advantages in respect to the use of live probiotics, mainly their safety profile, positioning them as interesting strategies for the management of common prevalent conditions in a wide variety of patients´ characteristics.

## Figures and Tables

**Table 1:** Advantages of inactivated bacteria and/or purified compounds in comparison with live probiotics.

Aspect	Advantages
Safety	No risk of translocation from gut lumen to blood, particularly in vulnerable subjects. No risk of acquisition and retransfer of antibiotic resistance genes. No risk of interference with normal colonization of gut microbiota in neonates.
Physiological effects	Release of active molecules from the disrupted inactivated cells, passing through the mucus layers and stimulating epithelial cells more directly. Loss of viability and cell lysis can produce further and more complex beneficial effects.
Pharmaceutical characteristics	Easier to standardize, transport, and store.

**Table 2:** Immunomodulating effects of heat-killed bacteria and cell-free supernatants.

Immunomodulating Properties			
Component/Fraction	Species	Effects	References
Heat-killed bacteria	*L. paracasei*, *L. reuteri*, *L. casei*, *L. plantarum*	Induction of IL-12	[38]
	Combination of *L. acidophilus*, *L. plantarum*, *L. fermentum*, and *E. faecium*	Enhanced immunomodulatory activity in comparison with live strains. Treatment at 100 °C for 30 min did not alter their adhesive capacity	[102]
	*S. thermophilus*	Production of IgA	[77]
	*L. rhamnosus* OLL2838	Barrier protective properties in mice with induced colitis	[103]
	*L. acidophilus* LB + culture medium	Reduced paracellular permeability	[104]
	Product containing *B. breve*, *B. longum*, *B. infantis*, *L. acidophilus*, *L. plantarum*, *L. paracasei*, *L. bulgaricus*, and *S. thermophilus*	Protection of cyto-architecture of intestinal barrier; down-regulation of TNF-α expression	[105]
	*B. breve* M-16-V	Suppression of pro-inflammatory cytokine production	[106]
	*B. bifidum* OLB6378	Increased expression of sIgA receptor	[107]
Cell-free supernatants	*L. acidophilus*, *L. casei*, and *L. reuteri*	Down-regulation of PGE-2 and IL-8 expression	[68]
	*L. delbrueckii*, *L. paracasei*, *L. salivarius*, *L. reuteri*, *L. rhamnosus*, *L. acidophilus*, *L. plantarum*, *L. lactis*, *L. casei*, *S. thermophilus*, *B. breve*, and *B. longum*	Anti-inflammatory responses mediated by metabolites and cell surfaces. Stimulation of cell-surface structures of PBMC similar to olive strains	[33]
	Soluble factors of *L. reuteri* CRL1098	Anti-inflammatory responses	[68,108]
	Soluble peptides of *L. rhamnosus* GG	Prevention of cytokine-induced cell apoptosis	[19,109]
	Metabolites from *B. breve*	Immunomodulation in human dendritic cells	[68,110]

**Table 3:** Protection against pathogens of heat-killed probiotic bacteria and cell-free supernatants.

Protective Effects against Pathogens			
Component/Fraction	Species	Effects	References
Heat-killed bacteria	*Lactobacillus*	Competition for adhesion sites (enterotoxigenic *E. coli* -ETEC-, *Campylobacter*, *H. pylori*	[59,89,111,112]
	Combination of *L. acidophilus*, *L. plantarum*, *L. fermentum*, and *E. faecium*	Reduction of *Salmonella* invasion and the induced inflammation	[102]
	*L. plantarum*	Protection against *Salmonella* infection and reduction of translocation	[113]
	*L. johnsonii*	Inhibition of *H. pylori* growth	[89]
	Bifidobacteria	Resistance to *Salmonella* infection	[114]
	*Bifidobacterium* BB12	Interference with *S. mutans* biofilm formation	[115]
Cell-free supernatants	Lactic acid bacteria	Release of bacteriocins, inhibition of Gram-positive and Gram-negative bacteria	[39,116,117]
	Bifidobacteria	Release of bacteriocins, against Gram-positive and Gram-negative bacteria and yeasts	[28,118,119]

## Abbreviations

EPS	Exopolysaccharides
NK	Natural killer
APCs	Antigen-presenting cells
Th1	Type 1 helper T
LPS	Lipopolysaccharide
TLR2	Toll-like receptor-2
NEC	Necrotizing enterocolitis
CDAD	*C. difficile*-associated diarrhea
TEER	Transepithelial electrical resistance
SIBO	Small intestinal bacterial overgrowth
UTIs	Urinary tract infections
